# Preparation and Application of High Internal Phase Pickering Emulsion Gels Stabilized by Starch Nanocrystal/Tannic Acid Complex Particles

**DOI:** 10.3390/gels10050335

**Published:** 2024-05-15

**Authors:** Haoran Jin, Chen Li, Yajuan Sun, Bingtian Zhao, Yunxing Li

**Affiliations:** 1 Key Laboratory of Synthetic and Biological Colloids, Ministry of Education, School of Chemical and Material Engineering, Jiangnan University, Wuxi 214122, Chinabtzhao@jiangnan.edu.cn (B.Z.); 2 School of Chemistry, Biology and Environment, Yuxi Normal University, Yuxi 653100, China

**Keywords:** starch nanocrystal, tannic acid, Pickering stabilizer, high internal phase emulsions (HIPEs), HIPE gels

## Abstract

Herein, the starch nanocrystal/tannic acid (ST) complex particles, which were prepared based on the hydrogen bond between starch nanocrystal (SNC) and tannic acid (TA), were successfully used to stabilize the HIPPE gels. The optimal TA concentration of the ST complex particles resulted in better water dispersibility, surface wettability, and interfacial activity as compared to SNC. The hydrogen bond responsible for the formation of ST complex particles and subsequent stable emulsions was demonstrated by varying the pH and ionic strength of the aqueous phase. Notably, the HIPPE gels stabilized via the ST complex particles can maintain long-term stability for up to three months. The HIPPEs stabilized via the ST complex particles all displayed gel-like features and had smaller droplets and denser droplet networks than the SNC-stabilized HIPPEs. The rheological behavior of HIPPE gels stabilized via the ST complex particles can be readily changed by tuning the mass ratio of SNC and TA as well as pH. Finally, the prepared HIPPE gels used to effectively protect encapsulated *β*-carotene against high temperatures and ultraviolet radiation and its controllable release at room temperature were demonstrated. It is anticipated that the aforementioned findings will provide new perspectives on the preparation of Pickering emulsion for delivery systems.

## 1. Introduction

High internal phase emulsions (HIPEs) are extremely concentrated emulsions with gel and emulsion qualities when the internal phase volume fraction is more than 74 vol%. Due to their huge surface area, traditional HIPEs are stabilized via high concentrations of surfactants, which may have negative impacts on the environment and human health [1]. Furthermore, their inadequate resilience against droplet coalescence and Ostwald ripening results in their poor stability. On the other hand, colloidal particle-stabilized high internal phase Pickering emulsions (HIPPEs) are more stable and biocompatible when compared to surfactant-stabilized HIPEs. Along with large surface area and adjustable viscoelasticity, HIPPE gels have a variety of potential applications, including delivery carriers [2], solid-like foodstuffs [3], catalyst supports [4], porous scaffolds [5], and 3D printing [6].

Although a variety of inorganic particles and polymer microgels have been shown to prepare HIPPE gels, their unsustainability and low safety make them unsuitable for applications in the food, cosmetic, and pharmaceutical industries [7]. Colloidal particles derived from biomass (proteins and polysaccharides) are becoming increasingly popular as stabilizers, replacing synthetic or inorganic particles for HIPPE gels due to their biocompatible, low-cost, and renewable properties. Pickering stabilizers of biological origins, such as gliadin nanoparticles, gelatin nanoparticles, starch/chitosan complex [8], myofibrillar protein microgels [9], Spanish mackerel protein-procyanidin hybrid nanoparticles [10], and so on, have been prepared and investigated as stabilizers for HIPPE gels.

Starch nanocrystal (SNC) is extracted from native waxy maize starch through acid hydrolysis and is recommended as a Pickering stabilizer [11]. SNC, however, performs poorly at emulsifying because of its intrinsic hydrophilic character and ease of aggregating and settling in water [12]. Considerable attention has been devoted to chemically modifying SNC through grafting [13] or crosslinking [14] to enhance its hydrophobicity and compatibility. However, these procedures lessen the sustainability and biocompatibility of SNC, especially when it applies to food, cosmetics, and pharmaceuticals. It is safe and sustainable to prepare food-grade complex particles as novel Pickering stabilizers through noncovalent interactions between naturally occurring biopolymers and small molecules. The FDA has recognized tannic acid (TA), a polyphenolic compound present in various plants, to be generally safe [15]. TA can combine with proteins or polysaccharides through a variety of molecular interactions. Despite the fact that TA has been widely incorporated into complex particles with proteins and subsequently used as stabilizers for HIPPE gels, there has been little research on the complexation of TA with polysaccharides and the employment of such complex particles in the preparation and stabilization of HIPPE gels.

Herein, we successfully prepared stable HIPPE gels by complexing SNC with TA to generate SNC/TA (ST) complex particles. The presence of TA allows HIPPE gels to have smaller droplet sizes and denser droplet networks, which are thought to be more stable, while also providing a functional interface and better protection against the lipophilic bioactive substances than SNC alone. In detail, the complexation of SNC with TA and the variation in interfacial properties of the resulting ST complex particles were first analyzed. The influence of TA on the emulsifying ability of ST complex particles was then verified by comparing the stability of emulsions prepared using SNC with TA or not. Subsequently, the influence of pH and ionic strength on the preparation of HIPPE gel were investigated to verify the interaction underlying the complexation between SNC and TA. Finally, *β*-carotene was selected as a model to investigate the protection against high temperatures and ultraviolet (UV) radiation and controlled release at ambient temperature of the resulting HIPPE gels for lipophilic bioactive substances.

## 2. Results and Discussion

### 2.1. Preparation and Characterization of ST Complex Particles

**Figure 1:**
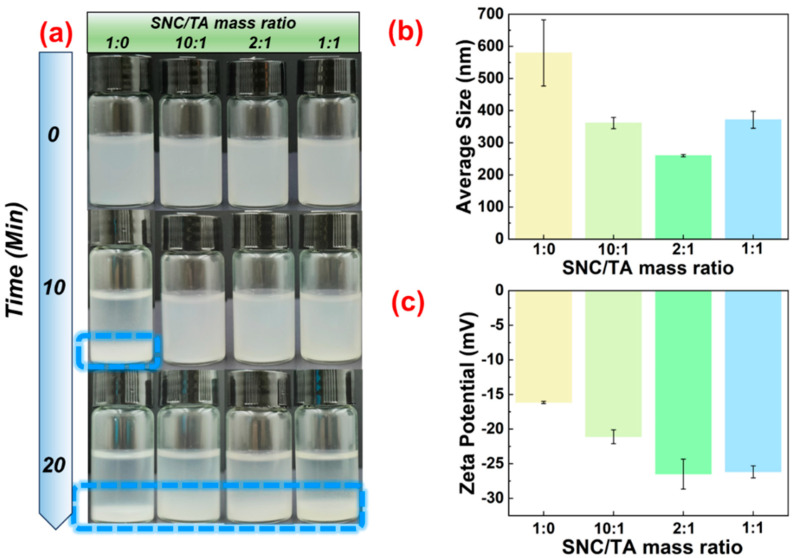
(**a**) Water dispersibility, (**b**) average size, and (**c**) zeta potential of SNC and ST complex particles at pH of 5; the concentration of SNC was 0.1 wt%.

To begin, the dispersion of SNC was mixed with the TA solution to form the ST complex particles. As shown in Figure 1a, adding TA significantly increased the water dispersibility of SNC. SNC settled fully within 10 min, whereas the sedimentation of the sample was observed after 20 min for mass ratios of SNC to TA of 1:1, though less prominent at lower mass ratios (10:1 and 2:1). These phenomena suggest that the addition of TA can improve the dispersion of SNC in water, but the amount of TA should be limited. Next, the influence of TA on the microscopic state and surface properties of SNC was investigated via dynamic light scattering (DLS) and zeta potential measurements. DLS studies revealed SNC to be about 579.5 nm, indicating a considerable degree of aggregation, because the SEM image in Figure S1 showed the average size of SNC was approximately 40–100 nm, comparable with previous reports [16,17]. Interestingly, the average size of samples fell at first and then increased as the TA concentration increased. This changing trend corresponded to the change in the sedimentation of the samples. The complexation between SNC and TA may be the primary cause of the above-observed results. The presence of complexation can be verified by the change in the zeta potential of SNC upon the addition of TA. In Figure 1c, the zeta potential of SNC at a pH of 5 was about −16.17 mV. This is because, following H_2_SO_4_ hydrolysis, they have carboxyl and sulfate esters on their surface. Notably, the zeta potential of SNC became more negative when the TA concentration increased. These changes in zeta potential further suggest the presence of complexation, in which TA adsorbs to the surface of SNCs, forming ST complex particles that cause noticeable changes in surface electrical properties. On the other hand, it can be used to explain changes in particle size and the dispersion of SNCs in water when TA concentrations vary.

**Figure 2:**
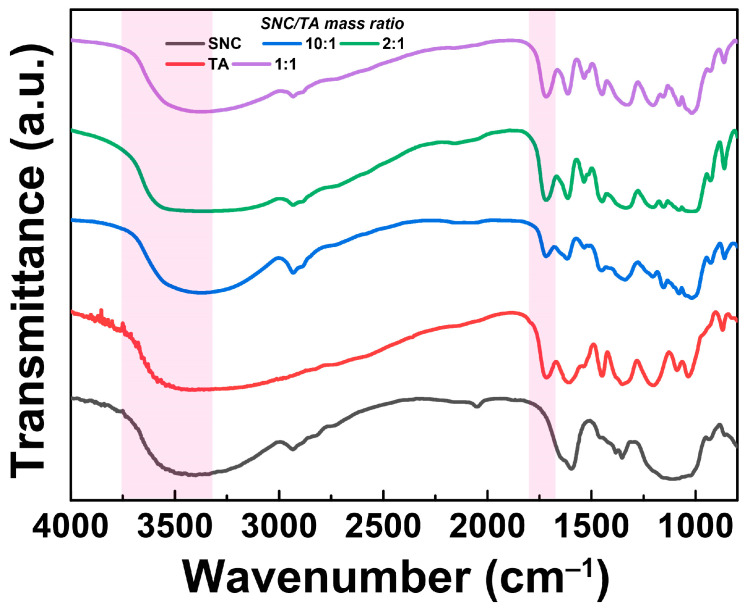
Fourier-transform infrared (FTIR) spectra of SNC, TA, and ST complex particles obtained from varying the mass ratios of SNC to TA.

With the aid of Fourier-transform infrared (FTIR) spectroscopy, the complexation between SNC and TA was explored. In Figure 2, the anti-symmetrical stretching vibration of -CH_2_ was indicated by a significant absorption peak seen in SNC at 2934 cm^−1^, while the C-O-O stretching vibration in a carbohydrate group was characterized by a peak at 1642 cm^−1^ [18]. As more TA was added, the broad band at about 3400 cm^−1^, which represented the O-H stretching vibration both in SNC and TA, shifted to lower wavenumbers, suggesting the hydrogen bond formed between them [19,20]. Furthermore, the -C=O vibration in TA increased somewhat from 1716 to 1718 cm^−1^, indicating that carbonyl groups in TA and hydroxyl groups in SNC formed a hydrogen bond [21]. No new absorption peak was observed in these FTIR spectra, indicating that non-covalent bonds rather than covalent ones had been formed between TA and SNC [22].

**Figure 3:**
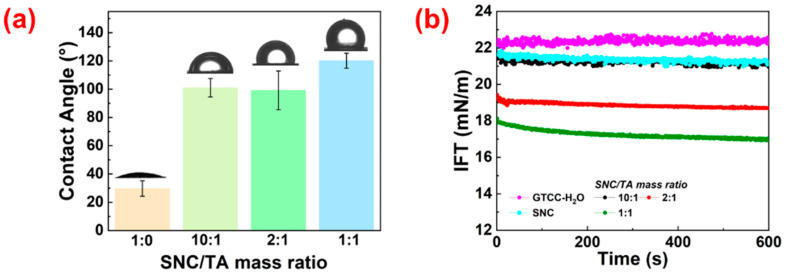
(**a**) Three-phase contact angle (*θ*_ow_) of a water droplet on sample tablets of SNC and various ST complex particles immersed in GTCC; (**b**) dynamic interfacial tension of oil/water interface with or without SNC and various ST complex particles.

An essential feature of colloidal particles is their three-phase contact angle (*θ*_ow_), which serves as a quantitative indicator of how wettable their surfaces are. Consequently, changes in *θ*_ow_ can reflect variations in the surface properties of colloidal particles. In Figure 3a, the *θ*_ow_ of a series of ST complex particles generated at various mass ratios of SNC and TA is displayed beside the *θ*_ow_ of SNC. As evidenced by the *θ*_ow_ of SNC approaching 30°, they are hydrophilic, corresponding to their abundance of surface hydroxyl groups. The coating of TA on the surface of SNC greatly raised the *θ*_ow_ of resulting ST complex particles. The complex particles produced at SNC/TA mass ratios of 10:1 and 2:1 had the *θ*_ow_ of about 100°, indicating their amphiphilic character. The *θ*_ow_ of ST complex particles further increased with the concentration of TA, approaching 120° at a mass ratio of 1:1 between SNC and TA. This increase in hydrophobicity was attributed to the coating of TA on the surface of SNC, which reduced the hydrophilic hydroxyl groups and increased the hydrophobic benzene rings exposed on the surface [23]. It was recognized that particle wettability is critical for the formation and stabilization of Pickering emulsions. Particles with intermediate wettability can be better wetted by both oil and water, resulting in greater adsorption energy and a robust barrier to prevent droplet coalescence [24,25]. The dynamic interfacial tension between oil and water in the presence of SNC and ST complex particles was examined further to study the difference in the interfacial behavior of these particles. At the GTCC/water interface, the equilibrium interfacial tension value was determined to be 22.35 mN/m. All other samples, except for SNC and ST complex particles (10:1), showed a decrease in interfacial tension over time. With a greater TA concentration, there was a greater degree of interfacial tension reduction. It was proposed that when TA is coated on the surface of SNC, the ST complex particles with increased interfacial activity are produced [26].

### 2.2. Stabilization and Characterization of Pickering Emulsions

**Figure 4:**
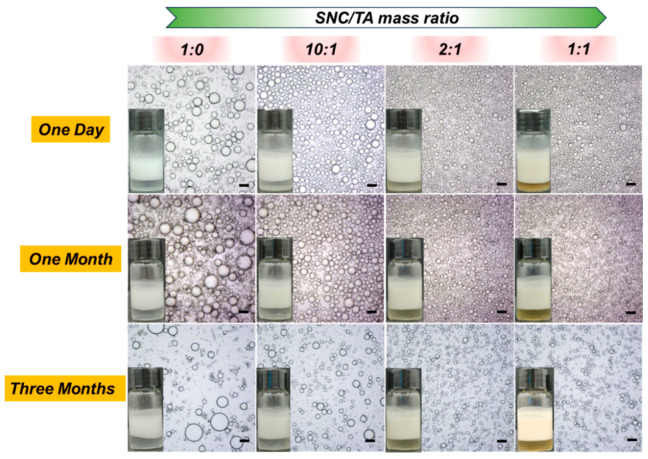
Visual appearance and optical microscopy images of Pickering emulsion stabilized with SNC and various ST complex particles after different storage times. The oil phase was GTCC with 50 vol% and the concentration of SNC was 1 wt%. Scale bars were 100 μm.

Figure 4 shows the formation and stabilization of Pickering emulsions using SNC and various ST complex particles as stabilizers. The largest droplet size was seen in the emulsion stabilized solely with SNC, and the droplet size clearly increased over a month. Even worse, after three months, a small amount of oil separated from the emulsion. However, compared to emulsions stabilized with SNC alone, the emulsions prepared with ST complex particles showed noticeably smaller droplet sizes, with the size decreasing continuously as the TA concentration rose. Remarkably, all emulsions stabilized with ST complex particles remained stable over time, exhibiting no evident variation in droplet size over three months, particularly when the mass ratio of SNC and TA surpassed 10:1. This suggests that TA additions, no matter how few, can significantly improve emulsion stability. The observed difference in emulsion stability was supposed to be explained by the difference in the surface wettability and interfacial activity of SNC before and after coating with TA, as previously established. When the mass ratio of SNC and TA exceeded 2:1, however, no appreciable change in droplet size and emulsion stability was noticed, and after creaming, the aqueous phase became yellow, indicating an excess of TA. Figure S2 illustrates the phase separation that occurred immediately upon preparation in an emulsion that included TA as a stabilizer, indicating its poor emulsification property. As a result, for the following experiments, the ST complex particles generated with an SNC/TA mass ratio of 2:1 were selected.

### 2.3. Influences of pH and Ionic Strength on the Formation of Pickering Emulsions

**Figure 5:**
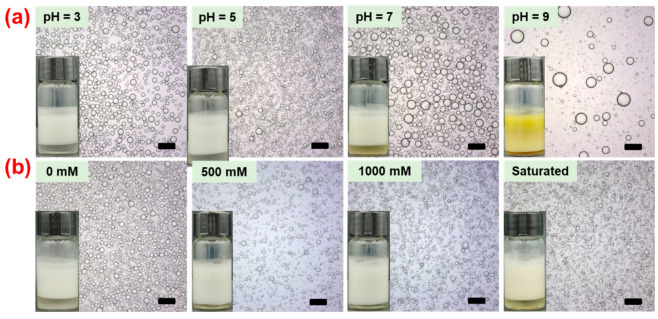
Visual appearance and optical microscopy images of Pickering emulsions stabilized with the ST complex particles with an SNC/TA mass ratio of 2:1 and kept at room temperature for one day. Experiments were carried out with varying pH (**a**) and ionic strength (**b**). The oil phase was GTCC with 50 vol% and the concentration of SNC was 1 wt%. Scale bars were 100 μm.

Emulsions are included in a variety of goods in the food, pharmaceutical, and cosmetics that must be subjected to varying conditions, such as varying pH and ionic strength, throughout manufacture and storage. It is crucial to comprehend the emulsion behavior in these circumstances. Hence, in the presence of SNC and TA, the influence of pH and ionic strength on the formation and stabilization of Pickering emulsions was examined. The droplet size of the resultant emulsions increased considerably with an increase in pH, especially when it was over 7, as seen in the optical microscope images in Figure 5a. Visual inspection showed that the oil leak happened after the emulsion prepared at a pH of 9 was left for one day. The hydrogen bond is commonly thought of as the result of a weak electrostatic attraction between a donor and an acceptor of a hydrogen bond [27]. Given that TA has a pKa of around 8.6, demulsification occurred at a pH of 9, indicating that TA deprotonation interrupted the establishment of hydrogen bonds with SNC and decreased the stability of prepared emulsions [28]. On the other hand, the droplet size in Figure 5b did not vary appreciably with increasing ionic strength, even at saturation concentrations of NaCl. This indicated that the ionic strength had a minimal influence on the preparation and stabilization of Pickering emulsion using both SNC and TA. Therefore, the non-covalent interaction between SNC and TA was dominated by hydrogen bonds rather than electrostatic interactions [29]. In other words, the stabilization of prepared Pickering emulsion is greatly aided by the formation of ST complex particles based on hydrogen bonds.

### 2.4. Preparation and Rheological Property of HIPPE Gels

**Figure 6:**
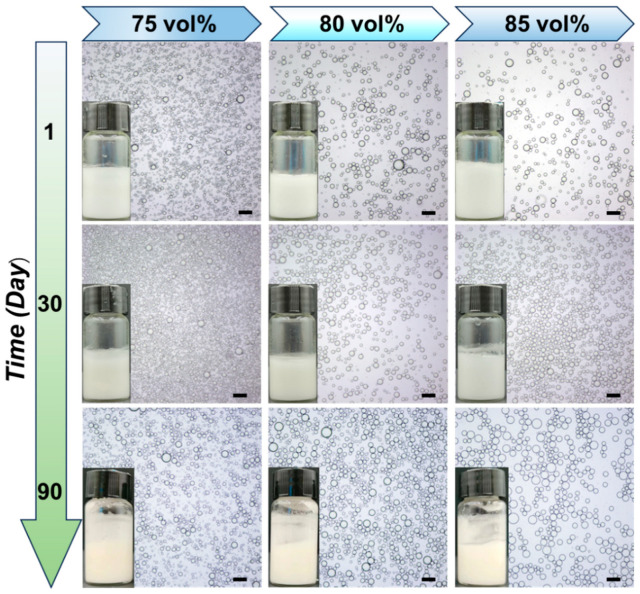
Visual appearance and optical microscope images of HIPPE gels stabilized with ST complex particles with an SNC/TA mass ratio of 2:1 and kept at room temperature for various periods. Scale bars were 100 μm.

Because of their superior emulsifying ability, further experiments were performed to examine the formation of HIPPE gels via the ST complex particles. Figure 6 shows the appearance and microstructure of Pickering emulsion stabilized with the ST complex particles, which have internal phase volume fractions ranging from 75 to 85 vol%. It was remarkable that, even after 30 days of storage at room temperature, these HIPPE gels stabilized with the ST complex particles remained homogeneous with no discernible signs of creaming and variation in droplet size. Even after three months, the resulting emulsions were stable against droplet coalescence, particularly at internal phase volume fractions of 75 vol%. Because the ST complex particles had adequate surface wettability and enhanced interfacial activity, they were able to stabilize the oil droplets directly and initiate the crosslinking of droplets, which resulted in a continuous network made up of oil droplets, leading to the formation of stable HIPPE gels.

**Figure 7:**
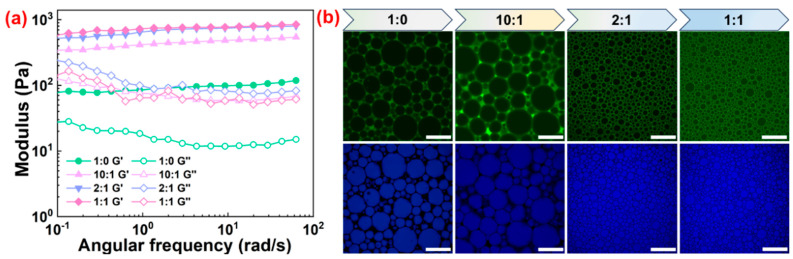
(**a**) Oscillation frequency sweep curves and (**b**) CLSM images of HIPPE gels (75 vol%) prepared with SNC and various ST complex particles at a pH of 5. The concentration of SNC was fixed at 1 wt%. Scale bars were 100 μm.

**Figure 8:**
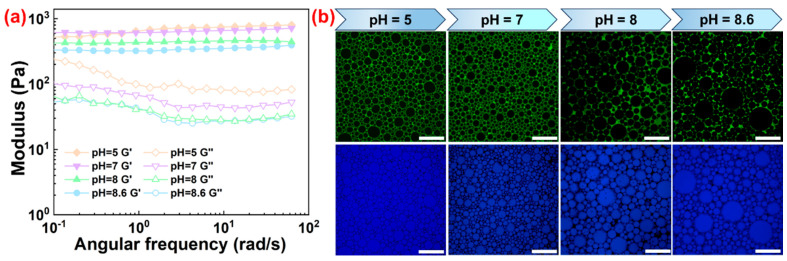
(**a**) Oscillation frequency sweep curves and (**b**) CLSM images of HIPPE gels (75 vol%) prepared at different pH using ST complex particles with a 2:1 SNC/TA mass ratio. The concentration of SNC was fixed at 1 wt%. Scale bars were 100 μm.

In their prospective applications, the rheological characteristics of HIPPE gels were essential for determining the correlation between process conditions and product quality. The rheological behavior of HIPPE gels (75 vol%) stabilized with SNC and various ST complex particles was first investigated. In Figure 7a, throughout the whole frequency range, the storage modulus (*G*′) of all emulsions was higher than the loss modulus (*G*″), demonstrating a classical gel behavior. As seen in Figure 7b, this behavior is primarily due to the close packing of oil droplets as well as the development of a network of ST complex particles surrounding the droplet surfaces [30]. Furthermore, compared to HIPPE gels stabilized with SNC alone, those stabilized with the ST complex particles showed greater *G*′ and *G*″. Additionally, when the mass ratios between TA and SNC increased, so did the *G*′ and *G*″ of the emulsions stabilized with the ST complex particles. This is due to the formation of a tighter internal crosslinked network structure between the smaller emulsion droplets inducing the enhanced interfacial interaction [31]. Therefore, the mass ratio of SNC/TA can be used to modify the rheological properties of HIPPE gels stabilized with the ST complex particles. On the other hand, there was also a close correlation between the pH and modulus of all HIPPE gels. Compared to HIPPE gels prepared at another pH, the one prepared at a pH of 5 showed greater *G*′ and *G*″ (Figure 8a). The droplet size of HIPPE gels became larger when the pH increased prior to emulsification (Figure 8b). The smaller oil droplets formed at a lower pH resulted in more intense droplet–droplet interactions. Moreover, as the ST complex particles adsorbed more effectively, the network structures they produced around the droplet surface became denser and stronger. Therefore, the results shown in Figure 8a suggest that adjusting pH alone can also modify the viscoelastic behavior of HIPPE gels stabilized with the ST complex particles.

### 2.5. Protection and In Vitro Release of β-Carotene

**Figure 9:**
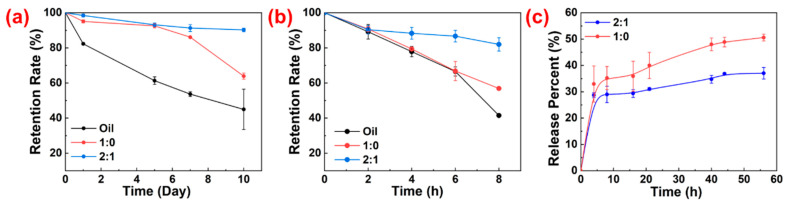
*β*-carotene retention in bulk oil and HIPPE gels (75 vol%) stabilized with SNC and ST complex particles at an SNC/TA mass ratio of 2:1 under (**a**) 50 °C and (**b**) UV; (**c**) in vitro release of *β*-carotene in HIPPE gels stabilized with SNC and ST complex particles at an SNC/TA mass ratio of 2:1 at room temperature, respectively.

The high stability and loading capacity of HIPPE gels make them advantageous for use as a vehicle for the protection and controlled release of lipid-soluble nutraceuticals or bioactive compounds such as *β*-carotene. Herein, a comparison was made between the protection of *β*-carotene in bulk oil and HIPPE gels stabilized with the ST complex particles and SNC alone. The findings in Figure 9a revealed that after 10 days, the retention of *β*-carotene in bulk oil dropped sharply to about 44.98% at 50 °C, while the retention in the two HIPPE gels stabilized with ST complex particles and SNC alone was about 90.21% and 63.90%, respectively. Furthermore, after 8 h of UV radiation, as shown in Figure 9b, the retention of *β*-carotene in bulk oil and the SNC-stabilized HIPPE gels was only about 41.47% and 56.89%, respectively. However, the retention was dramatically increased to about 81.99% when the ST complex particles were used to prepare the HIPPE gels. In summary, the HIPPE gels outperformed bulk oil in avoiding the degradation of *β*-carotene when exposed to high temperatures and UV radiation. Significantly, compared to HIPPE gels stabilized with SNCs alone, those stabilized with ST complex particles displayed greater protective capability. There are several reasons for these results. First, the *β*-carotene was better protected against pro-oxidants in the aqueous phase when there was a dense adsorption layer of particle stabilizer surrounding the oil droplets [32]. Second, the addition of TA reduced the exposed interface area to the aqueous phase because of the strengthened interfacial interaction between the emulsion droplets. Finally, the TA served as a UV filter and antioxidant, absorbing some UV radiation, chelating with metal ions in the aqueous phase, and eliminating radicals produced under various environmental stresses [31,33].

Next, using *β*-carotene as a model, the controlled release behavior of an encapsulated substance in the prepared HIPPE gels was studied. It has been demonstrated that the internal phase encapsulating substance diffused out through the gaps between absorbed particles at the interface [34]. The release curves for *β*-carotene from HIPPE gels stabilized with SNC and SNC/TA, which contained the same amount of *β*-carotene, are shown in Figure 9c. In the first 8 h, both samples showed a rapid release, and then the diffusion continued at a slow rate. Using SNC-stabilized HIPPE gels as vehicles, the initial rapid release was finished with about 35.2% encapsulated *β*-carotene. Nevertheless, during the same release period, the HIPPE gels stabilized with ST complex particles only released around 29.0% of the encapsulated *β*-carotene. The CLSM images in Figure 7b demonstrate the notable difference in droplet size and packing density between two HIPPE gels. It is believed that ST complex particles with improved wettability and interfacial activity may be better adsorbed, and, thus, a denser or thicker adsorption layer of particles forms, at the interface, slowing down the diffusion of the encapsulated *β*-carotene. Simultaneously, the oil/water interface area becomes smaller due to the tighter packing of smaller droplets, which also decreases the amount of substance diffused out at the same time. Consequently, it is noteworthy that the prepared HIPPE gels in this work have a higher superiority in the applications of food, cosmetics, and pharmaceuticals due to the easy tuning of the controlled release of lipophilic active compound from HIPPE gels by adding TA.

## 3. Conclusions

In summary, this work developed a new type of Pickering stabilizer by directly complexing SNC with TA via hydrogen bonding. The resulting ST complex particles can have better water dispersibility than SNC. The greater zeta potential of ST complex particles could be the cause of this. Furthermore, these complex particles exhibited a considerable improvement in hydrophobicity and effectively increased interfacial activity when compared to SNC. On the basis of these superior interface behaviors, long-term stable oil-in-water Pickering emulsions were prepared with success. Further evidence that the formation of complex particles based on hydrogen bonds was essential to obtain stable Pickering emulsions came from the influence of pH and ionic strength on emulsion preparation. Most importantly, these complex particles can be used to prepare HIPPE gels, all of which have semi-solid behavior. The pH and TA concentration can be adjusted to control the rheological behavior of the resulting HIPPE gels, which could be attributed to a decrease in emulsion droplet size, an increase in packing density, and an increase in the contact area between emulsion droplets. Lastly, the prepared HIPPE gels can successfully shield lipid-soluble substances from high temperatures and UV radiation while regulating their long-term gradual release.

## 4. Materials and Methods

### 4.1. Materials

Amylopectin was purchased from TCI (Shanghai) Development Co., Ltd. (Shanghai, China). Tannic acid (TA), soybean oil, and *β*-carotene were bought from Shanghai Macklin Biochemical Co., Ltd. (Shanghai, China). Sodium hydroxide, *n*-hexane, sulfuric acid (H_2_SO_4_), sodium chloride (NaCl), hydrochloric acid (HCl), and absolute ethanol were received from Sinopharm Chemical Reagent Co., Ltd. (Shanghai, China). Tween 80 was bought from Shanghai Titan Scientific Co., Ltd. (Shanghai, China). Caprylic/capric triglyceride (GTCC) was obtained from Croda. (Cowick Hall, UK). Fluorescein sodium was bought from Innochem Science & Technology Co. Ltd. (Beijing, China). Pyrene was purchased from Sigma-Aldrich. (Shanghai, China). All experiments were carried out with deionized water.

### 4.2. Preparation of Starch Nanocrystal (SNC)

The process of starch hydrolysis using acid was used, following the guidelines reported by Angellier et al. [35]. Briefly speaking, starch powder (20 g) and 150 mL of H_2_SO_4_ (3.16 M) were mixed and stirred at 200 rpm for one week at 40 °C. After that, the mixture was centrifugated and washed with water repeatedly until the pH of the supernatant did not change. The resultant dispersion of SNC (3 wt%) was stored at 4 °C.

### 4.3. Preparation of SNC/TA (ST) Complex Particles

A total of 1 mL of aqueous dispersion of SNC (3 wt%) was mixed with TA solution (3 wt%) at varying volumes. The concentration of SNC in the resulting mixture was adjusted to 1.0 wt% using deionized water. Additionally, the mass ratios of SNC to TA were established as follows: 1:0, 10:1, 2:1, and 1:1. After adding HCl (0.1 M) to bring the pH of the mixture to 5, they were vortexed for 2 min at room temperature.

### 4.4. Characterization of SNC and ST Complex Particles

The morphology of freeze-dried SNC was observed using an S-4800 scanning electron microscope at 2 kV (Hitachi, Japan). After dispersing the sample in water, a certain amount of resulting dispersion was dropped onto a tiny silicon wafer fragment. After drying at room temperature, the sample was coated with gold overlayer.

The zeta potential and particle size of SNC and ST complex particles were quantified using a ZetaPALS analyzer (Brookhaven, MA, USA). The pH of the aqueous dispersion of SNCs (0.1 wt%) was adjusted to 5 by HCl (0.1 M).

The contact angle of water droplets on the sample disc submerged in GTCC as well as the dynamic tension of the oil/water interface over time were quantified using the OCA 15EC (DataPhysics, Filderstadt, Germany) equipped with an image capture system. After the discs were placed in a colorimetric dish that was filled with GTCC, a water droplet (2.5 μL) was deposited on the surface of the sample. Following a minute of equilibrium, an image was taken and the Laplace–Young equation was fitted to determine the contact angle.

Fourier-transform infrared (FTIR) spectroscopy was conducted with a Nicolet iS50 spectrometer (Waltham, MA, USA) using samples prepared via the KBr disk method and scanning across the range of 400–4000 cm^−1^.

### 4.5. Preparation of Pickering Emulsions and High Internal Phase Pickering Emulsion (HIPPE) Gels

The dispersion of SNC (1 mL, 3 wt%) was mixed with varying volumes of TA solution (3 wt%), the concentration of SNC was diluted to 1 wt% using deionized water, and the pH was adjusted to 5 using HCl (0.1 M). The resulting mixture was vortexed for 2 min before the emulsion preparation. Following the addition of GTCC in an equal volume, an emulsion was formed after homogenizing at 13,500 rpm for 2 min. Under a fixed mass ratio of SNC/TA (2:1), the influences of pH, ionic strength, and oil phase volume fraction on emulsion preparation were evaluated. The pH varied from 3 to 9, the ionic strength varied from 0 to saturation, and the oil phase volume fraction varied from 50 to 85 vol%.

### 4.6. Confocal Laser Scanning Microscopy (CLSM)

CLSM images of HIPPE gels were captured with a Nikon Eclipse Ti2 microscope (Tokyo, Japan) with a 40× objective and laser lines of 405 and 488 nm. The aqueous and oil phases of the emulsion were stained with sodium fluorescein and pyrene, respectively.

### 4.7. Rheological Measurements

The rheology behavior of the prepared HIPPE gels was analyzed at room temperature using a Discovery HR-3 rheometer (New Castle, DE, USA) with a 2.004° cone plate of 40 mm and a gap height of 52 µm. The strain sweep was performed at a constant frequency of 1 Hz. The storage modulus (*G*′) and loss modulus (*G*″) were determined by increasing the frequency within the linear viscoelastic region from 0.1 to 100 rad/s.

### 4.8. Protection and In Vitro Release of β-Carotene

*β*-carotene (1 mg/mL) was dissolved in oil with the help of ultrasonication, and any undissolved *β*-carotene was removed via centrifugation. The Pickering emulsions containing *β*-carotene were prepared as described above. The resulting emulsions were kept in the dark at 50 °C for storage. The control group was consistent with the oil phase of the emulsion. To evaluate the stability under UV radiation, the glass bottle containing the emulsion was horizontally placed into a container with UV lamps (15 W) at room temperature. The retention rate of *β*-carotene was then measured every two hours.

The process used to extract *β*-carotene from the emulsions or control groups was modified based on the reported literature [36]. Typically, a sample (80 mg) was taken from the emulsion, mixed with deionized water (0.3 mL), ethanol (2 mL), and *n*-hexane (3 mL) continuously, and vortexed to facilitate the extraction of *β*-carotene. Next, the *n*-hexane was transferred to a brown volumetric flask. Following two extractions, the total volume of *n*-hexane in the brown volumetric flask was diluted to a constant volume (10 mL). The absorbance of each sample at 450 nm was measured three times using a UV–visible spectrophotometer.

Retention Rate = *C*_t_/*C*_0_ × 100%

where *C*_0_ and *C*_t_ represented the concentrations of *β*-carotene before and after storage.

In vitro release study of *β*-carotene was conducted based on the report by Tan et al. [37]. The corresponding HIPPE gels (1.0 g) were first placed on the bottom of glass bottles. The Tween 80 aqueous solution (4 mL, 3 wt%) and *n*-hexane (10 mL) were carefully added afterward. The bottles were shaken at 70 rpm at 25 °C, and the absorbance of the supernatants was periodically measured at 450 nm.

## Data Availability

All data and materials are available on request from the corresponding author. The data are not publicly available due to ongoing researches using a part of the data.

## Supplementary Materials

The following supporting information can be downloaded at https://www.mdpi.com/article/10.3390/gels10050335/s1: Figure S1: SEM image of the starch nanocrystals prepared with acid hydrolysis; and Figure S2: Macroscopic photograph of a freshly prepared emulsion using TA as a stabilizer.

## References

[1] Capron I., Cathala B. (2013). Surfactant-free high internal phase emulsions stabilized by cellulose nanocrystals. Biomacromolecules.

[2] Liu X., Xie F., Zhou J., He J., Din Z.-U., Cheng S., Cai J. (2023). High internal phase Pickering emulsion stabilized by zein-tannic acid-sodium alginate complexes: β-Carotene loading and 3D printing. Food Hydrocoll..

[3] Xu W., Li Z., Sun H., Zheng S., Li H., Luo D., Li Y., Wang M., Wang Y. (2022). High Internal-Phase Pickering Emulsions Stabilized by Xanthan Gum/Lysozyme Nanoparticles: Rheological and Microstructural Perspective. Front. Nutr..

[4] Guan W., Zhang Y., Wei Y., Li B., Feng Y., Yan C., Huo P., Yan Y. (2020). Pickering HIPEs derived hierarchical porous nitrogen-doped carbon supported bimetallic AuPd catalyst for base-free aerobic oxidation of HMF to FDCA in water. Fuel.

[5] Bizmark N., Du X., Ioannidis M.A. (2020). High Internal Phase Pickering Emulsions as Templates for a Cellulosic Functional Porous Material. ACS Sustain. Chem. Eng..

[6] Li X., Xu X., Song L., Bi A., Wu C., Ma Y., Du M., Zhu B. (2020). High Internal Phase Emulsion for Food-Grade 3D Printing Materials. ACS Appl. Mater. Interfaces.

[7] Dai L., Sun C., Wei Y., Mao L., Gao Y. (2018). Characterization of Pickering emulsion gels stabilized by zein/gum arabic complex colloidal nanoparticles. Food Hydrocoll..

[8] Xu T., Gu Z., Cheng L., Li C., Li Z., Hong Y. (2023). Influence of degree of substitution of octenyl succinic anhydride starch on complexation with chitosan and complex-stabilized high internal phase Pickering emulsions. Food Hydrocoll..

[9] Dai H., Sun Y., Feng X., Ma L., Chen H., Fu Y., Wang H., Zhang Y. (2023). Myofibrillar protein microgels stabilized high internal phase Pickering emulsions with heat-promoted stability. Food Hydrocoll..

[10] Zhou C., Zhang L., Zaky A., Tie S., Cui G., Liu R., Abd El-Aty A.M., Tan M. (2022). High internal phase Pickering emulsion by Spanish mackerel proteins-procyanidins: Application for stabilizing astaxanthin and surimi. Food Hydrocoll..

[11] Li C., Sun P., Yang C. (2012). Emulsion stabilized by starch nanocrystals. Starch-Stärke.

[12] Li C., Li Y., Sun P., Yang C. (2014). Starch nanocrystals as particle stabilisers of oil-in-water emulsions. J. Sci. Food Agric..

[13] Wang C., Pan Z., Wu M., Zhao P. (2014). Effect of reaction conditions on grafting ratio and properties of starch nanocrystals-g-polystyrene. J. Appl. Polym..

[14] Kim H., Park S., Lim S.T. (2015). Preparation, characterization and utilization of starch nanoparticles. Colloids Surf. B.

[15] Wang Y., Zhang J., Zhao Y., Pu M., Song X., Yu L., Yan X., Wu J., He Z. (2022). Innovations and challenges of polyphenol-based smart drug delivery systems. Nano Res..

[16] Kim H.Y., Lee J.H., Kim J.Y., Lim W.J., Lim S.T. (2012). Characterization of nanoparticles prepared by acid hydrolysis of various starches. Starch-Stärke.

[17] Haaj S.B., Thielemans W., Magnin A., Boufi S. (2016). Starch nanocrystals and starch nanoparticles from waxy maize as nanoreinforcement: A comparative study. Carbohydr. Polym..

[18] Ahmad M., Gani A., Hassan I., Huang Q., Shabbir H. (2020). Production and characterization of starch nanoparticles by mild alkali hydrolysis and ultra-sonication process. Sci. Rep..

[19] Wang J., Wang A., Zang X., Tan L., Ge Y., Lin X., Xu B., Jin Z., Ma W. (2018). Physical and oxidative stability of functional avocado oil high internal phase emulsions collaborative formulated using citrus nanofibers and tannic acid. Food Hydrocoll..

[20] Miao J., Xu N., Cheng C., Zou L., Chen J., Wang Y., Liang R., McClements D.J., Liu W. (2021). Fabrication of polysaccharide-based high internal phase emulsion gels: Enhancement of curcumin stability and bioaccessibility. Food Hydrocoll..

[21] Fan H., Wang L., Feng X., Bu Y., Wu D., Jin Z. (2017). Supramolecular Hydrogel Formation Based on Tannic Acid. Macromolecules.

[22] Zuo Y., Zhu F., Jiang S., Sui Z., Kong X. (2024). Structural, physicochemical, and digestive properties of starch-tannic acid complexes modulated by co-heating temperatures. Food Hydrocoll..

[23] Wei X., Li J., Eid M., Li B. (2020). Fabrication and characterization of emulsions stabilized by tannic acid-wheat starch complexes. Food Hydrocoll..

[24] Binks B.P. (2002). Particles as surfactants-similarities and differences. Curr. Opin. Colloid Interface Sci..

[25] Zou Y., Guo J., Yin S.W., Wang J., Yang X. (2015). Pickering Emulsion Gels Prepared by Hydrogen-Bonded Zein/Tannic Acid Complex Colloidal Particles. J. Agric. Food Chem..

[26] Dai H., Chen Y., Zhang S., Feng X., Cui B., Ma L., Zhang Y. (2021). Enhanced Interface Properties and Stability of Lignocellulose Nanocrystals Stabilized Pickering Emulsions: The Leading Role of Tannic Acid. J. Agric. Food Chem..

[27] Zhou J., Lin Z., Ju Y., Rahim M.A., Richardson J.J., Caruso F. (2020). Polyphenol-Mediated Assembly for Particle Engineering. Acc. Chem. Res..

[28] Li R., Zeng Z., Fu G., Wan Y., Liu C., McClements D.J. (2019). Formation and characterization of tannic acid/beta-glucan complexes: Influence of pH, ionic strength, and temperature. Food Res. Int..

[29] Zembyla M., Murray B.S., Radford S.J., Sarkar A. (2019). Water-in-oil Pickering emulsions stabilized by an interfacial complex of water-insoluble polyphenol crystals and protein. J. Colloid Interface Sci..

[30] Feng T., Fan C., Wang X., Wang X., Xia S., Huang Q. (2022). Food-grade Pickering emulsions and high internal phase Pickering emulsions encapsulating cinnamaldehyde based on pea protein-pectin-EGCG complexes for extrusion 3D printing. Food Hydrocoll..

[31] Liu Y., Yan C., Chen J., Wang Y., Liang R., Zou L., McClements D.J., Liu W. (2020). Enhancement of beta-carotene stability by encapsulation in high internal phase emulsions stabilized by modified starch and tannic acid. Food Hydrocoll..

[32] Xiao J., Li C., Huang Q.R. (2015). Kafirin Nanoparticle-Stabilized Pickering Emulsions as Oral Delivery Vehicles: Physicochemical Stability and in Vitro Digestion Profile. J. Agric. Food Chem..

[33] Li R., Tan Y., Dai T., Zhang R., Fu G., Wan Y., Liu C., McClements D.J. (2019). Bioaccessibility and stability of beta-carotene encapsulated in plant-based emulsions: Impact of emulsifier type and tannic acid. Food Funct..

[34] Dan N. (2012). Transport through self-assembled colloidal shells (colloidosomes). Curr. Opin. Colloid Interface Sci..

[35] Angellier H., Choisnard L., Molina-Boisseau S., Ozil P., Dufresne A. (2004). optimization of the preparation of aqueous suspensions of waxy maize starch nanocrystals using a response surface methodology. Biomacromolecules..

[36] Li Y., Wang R., Jiang H., Guan X., Yang C., Ngai T. (2022). Chitosan coated phytoglycogen for preparation of biocompatible Pickering emulsion. Colloids Surf. A.

[37] Tan H., Sun G., Lin W., Mu C., Ngai T. (2014). Gelatin Particle-Stabilized High Internal Phase Emulsions as Nutraceutical Containers. ACS Appl. Mater. Interfaces.

