# Supplementary material for: Preparation and Application of High Internal Phase Pickering Emulsion Gels Stabilized by Starch Nanocrystal/Tannic Acid Complex Particles
Source: Gels. 2024 May 15;10(5):335. doi: 10.3390/gels10050335 (PMC11121127; doi:10.3390/gels10050335)
Supplement: Supplementary file 1 [file gels-10-00335-s001.zip › gels-2989766-supplementary.pdf]

Preparation and Application of High Internal Phase Pickering Emulsion Gels Stabilized by Starch Nanocrystal/Tannic Acid Complex Particles

Haoran Jin [1,†], Chen Li [1,2,†], Yajuan Sun [1,*], Bingtian Zhao [1] and Yunxing Li [1,*]

[1] Key Laboratory of Synthetic and Biological Colloids, Ministry of Education, School of Chemical and Material Engineering, Jiangnan University, Wuxi 214122, China; btzhao@jiangnan.edu.cn (B.Z.)
[2] School of Chemistry, Biology and Environment, Yuxi Normal University, Yuxi 653100, China
* Correspondence: cmsun@jiangnan.edu.cn (Y.S.); yunxingli@jiangnan.edu.cn (Y.L.)
† These authors contribute equally to this work.

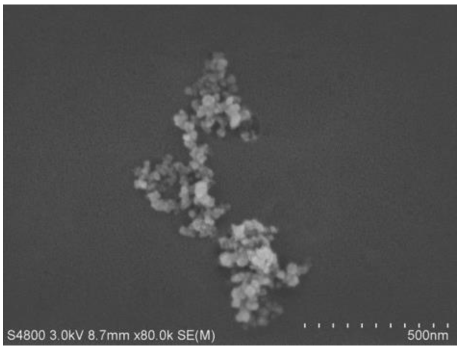

Figure S1. SEM image of the starch nanocrystals prepared with acid hydrolysis.

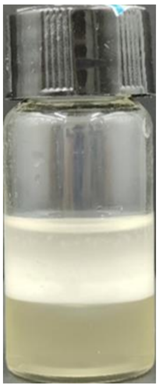

Figure S2. Macroscopic photograph of a freshly prepared emulsion using TA as a stabilizer.
